# Organizational Justice and Employee Readiness for Change: The Mediating Role of Perceived Organizational Support

**DOI:** 10.3389/fpsyg.2022.806109

**Published:** 2022-03-10

**Authors:** Senay Kebede, Aimin Wang

**Affiliations:** School of Management, Wuhan University of Technology, Wuhan, China

**Keywords:** organizational justice, overall justice, perceived organizational support, readiness for change, organizational change

## Abstract

Recent studies have shown that an organization must adapt to change to succeed in a constantly changing market. However, most change efforts fail due to employee resistance to change. It is critical to address employee readiness for change to avoid employee resistance. Employees’ perceptions of fair treatment by their organizations have positively predicted their Readiness for organizational change. This research aims to investigate the influence of organizational justice on employee readiness for change using perceived organizational support (POS) as a mediator. This study was carried out on the Ethiopian Revenue and Customs Authority (ERCA) and conducted with 359 employees. The study used a structural equation model and multiple regression analysis to analyze the data. The model developed explains how POS mediates the positive relationship between organizational justice and employee readiness for change. The result shows that organizational justice is a significant influencing factor on employee readiness for change. Furthermore, POS mediates the positive influence of organizational justice on employee readiness for change. This study can assist public and private organizations, as well as policymakers and practitioners, in improving and encouraging different organizational change practices in Ethiopia. Moreover, this study can also contribute to the literature on organizational change by filling the gaps in the relationship between organizational justice and employees’ Readiness for organizational change. Overall, this study concludes that organizations in Ethiopia, including ERCA, should investigate the influence of organizational justice on employee readiness for change to have successful organizational change.

## Introduction

The contemporary society is characterized by identification, confrontation, evaluation, and action – a process known as change (Paton, 2004). The need for continuous performance improvement, as well as the ongoing need to create new opportunities, are likely to drive change. Implementing successful change processes is essential to organizations’ survival (Paton, 2004; Deutschman, 2007). In the world we live in, change is a natural and universal process. As a result, change becomes necessary and unavoidable, as well as a method of adaptation and evolution for each individual, organization, and society (Popescu et al., 2012). Burnes (2004) agreed that an organizations’ competitiveness is no longer determined mainly by its production capacity or financial power but rather by its willingness to grasp change and innovate. Furthermore, researchers believe that organizations with effective change management strategies are more likely to survive and thus provide long-term employment for their employees. Change management is defined as the implementation and administration of initiatives that aim to renew an organization’s structure, direction, and capabilities to meet the needs of internal and external customers.

Employees are essentially responsible for implementing change initiatives, and change succeeds or fails based on employee behavior (Armenakis and Bedeian, 1999). The ability to forecast events and adapt to changing market conditions is critical for any organization’s long-term success. It is essential to respond to current market demands while remaining competitive, but market competitiveness is difficult to maintain (Belás and Sopková, 2016; Civelek et al., 2016; Korauš et al., 2017). Therefore, effective change management is critical for any organization that wishes to survive and thrive in today’s competitive environment. Employees are required not only to adapt to but also to accept change as a way of life as the business world becomes more complicated due to new technologies, methods, and procedures being developed. Employees must respond to continuous smaller-scale changes that occur almost daily and discrete large-scale change initiatives that significantly change how they do their jobs (Weick and Quinn, 1999). Employees must embrace new ways of doing things because of the technological “revolution” in the business world, which imposes dramatic changes in how they should accomplish their job duties. Although technological advancements promise significant increases in business efficiency and productivity, they may come at the expense of employee retention and satisfaction (Gilmore et al., 1997).

Understanding the behavioral and psychological roots of employee reactions to change is critical for understanding, managing, and supporting a major workplace transition. However, much of the research on organizational development and change focus on organizational-level change rather than individual-level change (Judge et al., 1999). Researchers recently proposed that people who are experiencing change can cope with change as well as the proclivity to resist change (Judge et al., 1999; Oreg, 2003). They hypothesize that attitudinal characteristics strongly influence change in reactions. Other studies have found that supervisor support and communication quality can significantly impact how employees perceive and respond to large-scale changes (Wanberg and Banas, 2000). Armenakis and Bedeian (1999) highlight the significance of work dealing with organizational members’ affective reactions to change implementations in reviewing the organizational change literature. These authors advocate for more empirical research into how most successfully implement change to minimize the costly consequences of negative employee responses to change. Change reactions are extremely complex, with aspects of the shift influencing the individual, work unit, and organizational levels. There is widespread agreement that the personal demands placed on employees because of change significantly impact their responses to change (Fedor et al., 2006).

Globalization and shifting ideologies contribute to the increased level of organizational change (Armenakis and Bedeian, 1999). Change is required for an organization’s people, processes, culture, and strategies to be integrated and aligned (Asfaw, 2017). Change becomes an unavoidable and an essential component of organizational life because it determines the long-term achievement and continued existence of organizations. Despite this, nearly two-thirds of all organizational initiatives to ensure the planned change are unsuccessful (Rafferty et al., 2013). Employee fewer perceptions about change is a major contributor to the failure of change initiatives (Armenakis and Harris, 2009). To deal with this, employees in organizations must be ready for change. The main question for change agents is how to prepare their organization’s employees for change (Choi, 2011). The commitment and ability of an organization’s members to implement organizational changes are referred to as its Readiness (Walinga, 2008). The cognitive precursor to employees’ opposition or acceptance for change efforts has defined as “readiness for change” (Weiner et al., 2009). Readiness for change refers to employees’ opinions, perceptions, and motivations about the required changes, as well as the organization’s ability to successfully implement those changes (Armenakis et al., 1993). Employee perceptions of fair treatment by their organizations are referred to as organizational justice (Wang et al., 2015). Justice is an individual assessment of organizational decisions and decision-making procedures (Adams, 1965). Before implementing organizational change, organizations must first understand an employee readiness for change based on existing organizational overarching justice. Organizational Justice emphasizes managers’ decisions, perceived fairness, and the relationship between employees and their surroundings in organizations. Enforcing policies, rules, and procedures for employees may create a sense of injustice/unfairness, leading to workplace problems (Sert et al., 2014).

Ethiopia has increased the implementation of change management activities since introducing a result-based management system in 1994. It was also developed using Business Process Reengineering, the Balanced Scorecard, and ISO standards. However, most of these change reforms are ineffective. Change reforms such as change management and organizational performance (Fetiya, 2015), and implementation of the business process reengineering in the public organization and higher education institutions (Debela, 2009; Dereje, 2010; Sibhato and Singh, 2012), was not successful due to various reasons. Some of these reasons were; organizational resistance, insufficient Readiness for change, problems related to creating a culture for change, lack of trust between management and employees, underestimating the role of politics in change, low organizational support, insufficient planning for change, and compressing the time needed for change. Furthermore, lack of training and education, problems with change resources, ineffective change leadership teams, problems with information technology investment and sourcing decisions, low organizational justice, and misperception about change all contributed to the failure of organizational changes.

Ethiopian Revenue and Customs Authority (ERCA) problems stemmed mainly from inadequate employee readiness for change and low organizational justice (Bekele, 2015). Even though ERCA implemented some change management reforms, it focused on evaluating change management tools such as the evaluation of the business process reengineering (Debela, 2009; Sibhato and Singh, 2012) and implementation of Balance scorecard (Fetiya, 2015). They have little attention to organizational justice and employee readiness for change. Regardless of the fact, one study found that distributive and procedural justice influence readiness for change (Shah, 2011); more research has been needed to determine the impact of overall justice on employee readiness for change. Justice researchers have echoed this point by advocating for further research on overall justice (Ambrose and Schminke, 2009). There has been little empirical research on the effect of organizational justice on change readiness (Rodell and Colquitt, 2009).

Significant theoretical improvements and empirical evidence support the critical mediating effect of perceived organizational support (POS) on organizational justice and employee readiness for change. However, according to the authors’ knowledge, no sufficient research has been conducted to investigate the role of POS in mediating the relationship between organizational justice and employees’ Readiness for change. Moreover, the previous contextual studies’ finding has inconsistencies, methodological, and a variable disparity with different arguments. So that, those limitations have inspired authors’ to investigate the influence of organizational justice on employee readiness for organizational change using POS as a mediator variable. Accordingly, this research aims to investigate predictors of change readiness by addressing two critical research questions that have received insufficient focus, such as

(1) This study assesses the impact of organizational justice on employee readiness for change through examination of change readiness dimensions.
(2) This study explores the effect of POS as a mediator in the overall justice-readiness for organizational change. Therefore, this research finding tried to address the above basic research questions aligned with the specified aims of this study. Besides, the study also identified and supported the expected theoretical and managerial implications or significance.

This study contributes to the literature in the following unique ways. Firstly, the study investigates the relationship between organizational justice and employee readiness for change. This study argues that organizational justice and employee readiness for change have positive relationships. Secondly, this study approved the positive relationship between organizational justice and POS. Thirdly, this study also explores the mediating effect of POS between organizational justice and Readiness for change and argues that POS mediates the positive relationship between organizational justice and Readiness for change. To test our arguments, we used the questionnaire data from the ERCA.

Furthermore, the researcher assumes that the findings of this study would better help ERCA managers to understand their employees’ reactions and the factors that affect their employees’ attitudes toward the change. The study would also have a paramount significance for policymakers. So that appropriate policies would be designed to encourage different organizational changes in Ethiopia. Moreover, the study would be greatly important in expanding the knowledge frontier of effective organizational changes globally. In addition, it will serve as an important material other researchers can use for their further studies.

## Literature Review and Hypotheses Development

This study reviews the literature on organizational justice, employee readiness for change, and the mediating role of POS based on relevant research. Furthermore, it gives significant attention to employee readiness for organizational change.

### Organizational Justice and Employee Readiness for Change

Change is concerned with resolving the organization’s problems and challenges to ensure its long-term growth and survival. The majority of organizational challenges and problems are the result of external and internal pressures that can have an impact on employee efficiency and organizational development. Change is driven by external and internal forces associated with business expansion or the need to respond to challenges (Barnett and Carroll, 1995). Change can be chaotic and dramatic because of the transition from a known to an unknown situation (Gleick, 1987; Abrahamson, 2000). Employees of the organization are the most affected in that situation, as they can develop various thoughts, feelings, and behaviors in response to the unknown crisis (Cummings and Worley, 2014).

Previous research has found that organizational justice positively impacts employees’ Readiness to change. For example, Employees’ low perceived fairness in the workplace creates an unpleasant situation and increases negative emotional reactions such as exhaustion, stress, and anger (Deyreh, 2012). Fair organizational justice, on the other hand, was linked to higher levels of acceptance, readiness, and commitment to organizational change (Korsgaard et al., 2002). According to justice research, when employees believe they are being treated fairly (Lee et al., 2021), they are more likely to develop attitudes and behaviors that support the successful implementation of change. Furthermore, higher levels of justice are associated with greater adaptability, acceptance of change, cooperation with change, and satisfaction with change (Dent and Goldberg, 1999; Piderit, 2000). According to Arnéguy et al. (2021), having fair overarching organizational justice will lead to employees having a positive perception of change, which will increase their Readiness for change. Empirical research such as (Marzucco et al., 2014; Arnéguy et al., 2018; Hussain et al., 2019; Arif et al., 2020) has consistently demonstrated the positive influence of organizational justice on change-related reactions.

Literature encourages employees to have diverse life experiences, motivation levels, knowledge, attitudes, and behavioral patterns (Ilgen and Pulakos, 1999). Organizational managers are concerned about how to survive in the future and remain competitive in the face of employee importance and often-unknown challenges. They advocate for changing organizational policies, strategies, and approaches in response to changing circumstances (Gleick, 1987; Abrahamson, 2000). Employees’ attitudes and behaviors toward an organizational change can be positive or negative (Lines, 2005). Employees’ positive reactions to change are possible because of their level of involvement (Armenakis and Bedeian, 1999). There has been a lot of research done in change management over the last few decades (Cunningham et al., 2002; Vakola et al., 2004; Madsen et al., 2005; Rafferty and Simons, 2006; Holt et al., 2007; Erturk, 2008). Some of them, such as organizational commitment, social relations at work, Change efficacy, and job satisfaction, contribute to the success of change programs (Rafferty and Simons, 2006). Individuals’ perceptions of fairness within organizations are referred to as organizational justice (Foster, 2010). Employees know that fair procedures and outcomes are more likely to accept organizational change. Fairness is important in developing positive attitudes and behaviors in an unknown situation created by change (Adams, 1965).

Traditionally, researches indicate that organizational justice has four main dimensions: procedural, distributive, interpersonal, and informational. According to some academics, justice dimensions do not reflect an individual’s workplace justice experience (Colquitt et al., 2013). Despite empirical evidence linking each type of justice to various employee attitudes and behaviors, little has known how these dimensions interact with others’ justice experiences. As a result, they advocated for a change in focus toward a more comprehensive assessment of justice (Greenberg, 2001; Shapiro, 2001). Overall justice is a general assessment of the fairness of an entity based on one’s perceptions and the perceptions of other members of the group. Similarly, the Fairness heuristic theory proposes that employees use a cognitive heuristic called “a general impression of fair treatment.” (Lind and Van den Bos, 2002). This theory provides a useful conceptual basis for describing the impact of organizational justice in guiding employees when confronted with organizational change. Several empirical studies have found evidence that justice plays a role in shaping people’s reactions to change, including Change-Readiness (Shah, 2011). However, two studies have investigated the role of fairness in change management (Rodell and Colquitt, 2009; Marzucco et al., 2014). Given the significance of organizational change readiness, many conceptual definitions have been proposed. However, the majority of them tend to focus on similar dimensions.

Readiness for change is regarded as a “cognitive precursor to either resistance or support for a change effort.” In this framework, change readiness is divided into four dimensions. These are appropriateness, management support, self-efficiency, and personal benefits (Holt et al., 2007). Appropriateness of change refers to an employee evaluating the change’s suitability for addressing organizational problems. The proposed change is deemed appropriate if individuals believe it is the best solution to its problem. Employees of an organization may value a planned organizational change because they think that change is needed. They may also appreciate it because they believe the change will effectively address a critical organizational issue.

Managerial support refers to the support of top decision-makers and managers to their employees. It is the responsibility of change agents and managers to foster more positive attitudes and perceptions. Employees’ perceptions of their ability to implement the proposed change have been defined as change self-efficiency. Having a high level of self-efficiency is associated with being open to change (Wanberg and Banas, 2000), increased participation (Frank, 2002) and commitment (Herold et al., 2008). Employees who are confident in their ability to understand and improve can perceive organizational change as an opportunity to improve their abilities. On the other hand, employees who are uncertain about their ability to learn and improve may perceive organizational change as a threat (Vithessonthi and Schwaninger, 2008).

In addition, personal values must be considered during organizational change. When organizations plan for change, it is critical to consider the rewards employees demand, such as money, benefits, and new opportunities. Employees who have intrinsically motivated perceive their work environment to be fair compared to employees who are uninterested in their task (Hannam and Narayan, 2015). As a result, if something is relevant to them, employees will be much more motivated to support the new changes and assist in making them work properly (Hultman, 2018). Given the growing evidence that justice influences employees’ behaviors and attitudes toward change, it is acceptable to expect overall justice associated with employee readiness for change. Furthermore, research into this association’s mechanisms is limited (Fuchs and Edwards, 2012). We can explain the effects of justice using social identity and social exchange theories (Kurtessis et al., 2017).

The importance of understanding employee motivation and its relationship to organizational change is emphasized by social exchange theory (Blau, 1964). Such organizational behavior approaches consider employees’ motivation to perform specific tasks within the context of mutual obligations between employees and organizations (Aselage and Eisenberger, 2003). Employees develop feelings of responsibility or dissatisfaction with their organization when they perceive favorable or unfavorable treatment. In other words, positive work experiences shape employees’ perceptions of organizational support. The Fairness heuristic theory’s central premise is that people use their perceptions of justice to determine whether they cooperate with authority over them. It is reasonable to believe that when employees are treated fairly, they will develop positive attitudes and behaviors. Employees will accept change if they are treated fairly and given appropriate methods, mechanisms, and procedures for achieving results.

**Hypothesis 1 (H_1_):** Organizational justices positively influence employee readiness for change.

### Organizational Justice and Perceived Organizational Support

Perceived organizational support is important for observing the social exchange between employees and their organizations. It refers to employees’ general beliefs about how much their contribution is valued and how the organization is concerned about their well-being (Eisenberger and Stinglhamber, 2011). POS is a contextual variable that can influence a change initiative’s success (Eisenberger et al., 1986). Employee attitudes and behavior are influenced by how they perceive their work situation during organizational change. Employees may oppose or support the change depending on the level of support within the organization (Coghlan, 1993). POS is linked to various workplace attitudes and outcomes (Eisenberger et al., 1990). Positive discretionary activities can increase POS. Employees evaluate and respond to injustice and justice in their organizations while keeping the fundamental concerns for understanding organizational behavior in mind (Mollahosseini et al., 2012).

Injustice in rewards, compensation and treatment of employees affects the performance of both the employees and the organization (Ferwerda, 2011). According to an organizational support theory, favorable and discretionary care by the organization increases POS for employees’ well-being. Employees may feel valued because of interactive justice, which draws attention to the importance of developing a mentally satisfying relationship with their organization. A supportive organization may recognize employees who have received credit for their contributions, and this appreciation strengthens their perception of organizational membership. One of the primary motivations for employees to put in more effort on the job is to demonstrate their worth for promotions and career advancement (Epstein and Ward, 2006). Employment security refers to providing employees with a regular and permanent job position rather than short-term contracts; these permanent contracts demonstrate an organization’s commitment to employees, which serves as an effective incentive (Fulmer and Shaw, 2018). Compared to POS, organizational justice is a sense of fairness toward the organization’s employees.

The concept of distributive justice developed from equity theory, which refers to employees’ perceptions of the fairness of the outcomes they receive. Employees who believe in fair outcomes distribution among all organization members are more likely to participate in different behaviors. Equitable outcome distribution has emerged as a key indicator of job success and effectiveness (Cho and Kessler, 2008). Procedural justice is also a strong predictor of organizational support. DeConinck (2010) discovered that procedural justice predicts POS. Procedural justice viewed as an essential resource in social exchange in the organizational context; it influences employees’ perceptions of their organization’s exchange relationship (Loi et al., 2006). Procedural justices demonstrate the organization’s consideration for employees’ rights, which contributes significantly to POS. Interactional justice predicts POS. Interactive justice shows the degree to which employees are treated with respect and dignity by authorities in the elaboration and implementation of procedures (Bies and Moag, 1986). Employees may perceive the organization’s support in this context because interactive justice may lead employees to believe that the organization appreciates their contributions and well-being, thereby attracting their focus to developing and maintaining a mentally satisfying relationship with that organization (Cheung and Law, 2008).

**Hypothesis 2(H_2_):** Organizational justices positively influence POS.

### Perceived Organizational Support as a Mediator in Relationship Between Organizational Justice and Employee Readiness for Change

Some studies deal with POS on individual change readiness. For example, social support is linked to feelings of control during change (Vardaman et al., 2016), implying that other types of support, such as organizational support, may also contribute to positive change outcomes. This claim is supported by research that links managerial support and readiness for change (Kirrane et al., 2017). According to Self et al. (2007), POS is associated with positive feelings toward change directives, implying that it may also foster Readiness. POS has been linked to increased risk-taking comfort (Neves and Eisenberger, 2014), indicating that it provides greater psychological safety when individuals are confronted with uncertainty, such as during organizational changes. Individuals reciprocate the support they receive (Gouldner, 1960; Blau, 1964), which may influence their likelihood of preparing for organizational change in response to receiving support; individuals with higher levels of POS may reciprocate by supporting organizational change initiatives. Furthermore, some studies show that POS affects organizational justice, e.g., organizational support may lead to a more positive perception of the legitimacy of organizational change among employees (Rousseau and Tijoriwala, 1999). This relationship of POS with organizational justice and Readiness for change suggests that POS significantly influences organizational justice and Readiness for organizational change. Based on this, the authors are inspired to evaluate the role of POS in mediating the explained variables.

Employees’ perceptions of justice are more likely to foster organizational support. Members of an organization who viewed their working environment as generally unsupportive were more likely to react cynically, have negative feelings, and eventually reject the change (Kiefer, 2005). According to the social exchange theory and the reciprocity norm, employees’ perceptions of organizational support influence their sense of obligation to their organization. There is a large body of evidence indicating that justice improves POS (Köhler et al., 2015). However, previous research has concentrated on linking specific justice aspects and POS. Overall justice may affect POS because it reflects a general view of the organization’s fairness.

Evidence from the social exchange literature suggests a link between POS and change readiness. When employees feel assisted, they feel obligated to contribute to their organization by enacting the reciprocity norm (Gouldner, 1960). They are more likely to contribute to the success of their organization (Babic et al., 2015). Organizational support facilitates a sense of duty to the organization (Köhler et al., 2015) and good behaviors at work. The development of the change recipient’s positive or negative feelings toward change is a successful response to a proposed change (Mossholder et al., 2000). The behavioral reactions of change recipients in a change effort can range from actively supporting implementation to opposing it (Stensaker and Meyer, 2012). POS fosters employee trust to improve organizational effectiveness (Argyris, 1962). Individual perceptions are linked to the support or commitment of management or leadership to the changes that will be implemented. Employees gain confidence in management when they see that the organization is committed to and supports the implementation of planned changes. As a result, employees will follow the organization’s change management plans. Employees believe that the organization’s reasons for making changes are logical. As a result, they focus on the benefits of change in the organization, the degree of change efficiency achieved, and the compatibility of organizational goals with change goals. Employees who believe they have the knowledge, skills, and abilities to carry out their responsibilities will act following what the organization desires, including the desire to implement change. Employees’ self-confidence is essential for the successful implementation of planned changes.

Several studies have found that employees’ resistance to changes is exacerbated by their mistrust of those driving the change. Therefore, managers or leaders must support their employees when considering an organizational change. Justice has regarded as the cornerstone of social exchange in organizational settings (Organ, 1990; Colquitt et al., 2001; Cropanzano et al., 2001; Swalhi et al., 2017). Employees will feel supported by the organization if they see justice, which fosters a sense of obligation to repay the organization through positive feelings. Employees are more likely to accept change if they believe their organization supports them. These perceptions of organizational support can instill confidence in employees that, despite the changes, they are valued members of the organization. Such emotions may increase an employee’s perception that the organization is looking out for their best interests while implementing change, fostering employee readiness for change. The perceptions and affective sentiments elicited by POS may lead to an employee’s desire to reciprocate support by developing greater change readiness.

**Hypothesis 3 (H_3_):** POS mediates the positive relationship between organizational justice and employee readiness for change.

### Conceptual Model of the Study

**FIGURE 1:**
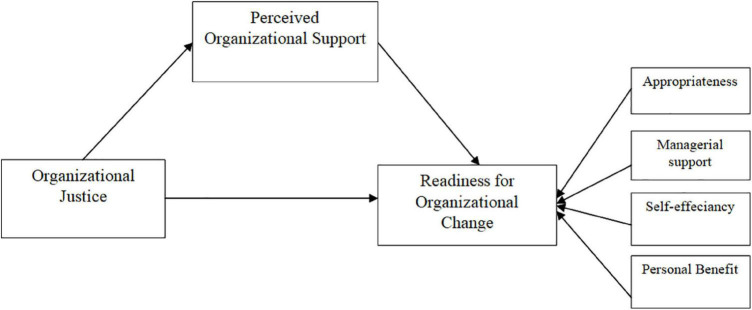
The study’s proposed research model.

The study proposed a research model to evaluate the influence of organizational justice on employee readiness for change using POS as a mediator. This model incorporates the independent variable organizational justice, the mediator variable POS, and the dependent variable employee readiness for organizational change. Appropriateness, managerial support, self-efficiency, and personal benefit dimensions are indicators of employee readiness for change. Thus, based on existing literature, we propose the following conceptual research model of the study depicted in Figure 1.

## Materials and Methods

### Sample and Data Collection

All ERCA employees in Ethiopia are included in the study population. The total number of employees across the 20 offices is 7370. The probabilistic sampling method (Kumar, 2011), specifically stratified random sampling, was used by the author. Different ERCA branches were classified into different strata based on similarities in the type of customer they are intended to serve. The assumption underlying the use of customer type as the basis for stratification is related to the likely difference in the process employed based on the different needs of customers. Because of the homogeneity between branches, only one branch was chosen randomly from each stratum. Following branch selection, participants from each office were determined using a simple random sampling technique; thus, each member of the population had an equal chance of being chosen. The researcher used the sample size determination formula developed at University Park (Watson, 2001).

$$\text{n}=\frac{\left(\frac{p\,\left[1-p\right]}{\frac{A^{2}}{Z^{2}}+\frac{p\,\left[1-p\right]}{N}}\right)}{\text{R}}\;\mathrm{Source}:\text{Watson},2001.$$

*N* = the total number of people in the population = 7370; *n* = required sample size = 373; P = population variance estimation = 50%; A = desired precision = 5%; Z = Based on confidence level = 95%; R = Response rate estimated = 98%.

Using the formula mentioned above, the author chose 373 participants at random from a total population of 7,370 employees of the branches selected to collect relevant data. Furthermore, proportional stratified sampling was used in this study. The 373 respondents were drawn at random from each branch. Because the number of employees in each branch differs, the sample numbers for each branch were computed as follows in Table 1.

**TABLE 1:** Sampling determination.

S. No	Cluster name	No. branches	Selected branch	No. employees	Proportion (%)	Sample selected
1	Head office	1	Head office	1066	[(1066/7370) x 373] = 54	54
2	Kuha	1	Kuha	328	[(328/7370) x 373] = 17	17
3	Semen	4	Semen 02	228	[(228/7370) x 373] = 30	12
4	Qedemai weyane	10	Qedemai weyane 03	3833	[(3833/7370) x 373] = 194	194
5	Ayder	1	Ayder	642	[(642/7370) x 373] = 33	32
6	Hawelti	1	Hawelti	699	[(699/7370) x 373] = 36	35
7	Adi-haki	2	Adi-haki 01	574	[(574/7370) x 373] = 29	29
Total						373

The research designed quantitative approaches as per the aims of the study. The study also used primary and secondary sources of data. The primary data sources have been generated from employees’ responses using distributed questions. The secondary data has been incorporated from various reports and documents of the organization. Furthermore, the researcher contacted the organization’s leaders before developing the questionnaires and during the early stages of the survey for two major reasons:

(1) The researcher meets with the organization’s leaders to discuss the organization’s challenges in implementing changes. In addition, to ensure employees’ participation in research.

(2) The researcher meets with organizations’ leaders to obtain permission to use and referee various documents, reports, brochures, and other templates.

After an extensive review of related literature and consultation with the institution’s managers and staff, the questionnaires were developed using the research’s conceptual framework. Each section of the questionnaire items was created to address a different research variable. After a few weeks of initial contact, the researcher provided data collection instructions. As a result, the researcher made certain that every employee was aware of the survey’s voluntary and anonymous nature. For this study, 373 respondents have targeted, with 359 completing the entire questionnaire, yielding a response rate of 96.2%. In preparation for analysis, data have been checked, labeled, and entered into SPSS software version 25 and AMOS software version 23.0.

### Measurement Items

In this research, the authors used adopted measurement instruments from previously validated research findings to evaluate both the predictor and outcome variables. Moreover, all the selected respondents were asked to rate their level of agreement with each measuring item through a five-point Likert scale instrument ranging from 1 = strongly disagree to 5 = strongly agree.

### Organizational Justice

Data on organizational justice was gathered using the overall justice measure developed by Colquitt (2001). This composed scale had eight items. Examples of items include, “Employees have the right to question or appeal job decisions made by their managers.”

### Perceived Organizational Support

Data on POS was gathered using the POS measure developed by Rhoades and Eisenberger (2002). This scale had six items, all of which conceptualize organizational support. Examples of items include “My organization values my contribution to its success.”

### Employee Readiness for Organizational Change

Data on employee readiness for change was collected from employees’ Readiness for organizational change measures developed by Vithessonthi (2005). This composed scale had six items. Examples “I am supported too, or will support this change.” and “On this change, I have fully cooperated with the organization.” employee readiness for organizational change has also been checked through the following dimensions.

### Change Appropriateness

Data on change appropriateness was gathered using the appropriateness of change measure developed by Holt et al. (2007). There were three items on this composing scale. Examples of items include “I completely understand why this change is required.”

### Managerial Support

Data on managerial support was gathered using managerial support to employee measures developed by Holt et al. (2007). This composed scale had three items. Example “I believe the motives and intentions of top management for their employees are good.”

### Self-Efficiency

Data on self-efficiency has been gathered utilizing the employee’s self-efficiency measure developed by Holt et al. (2007). This composed scale had three items. Examples of an item include “I am confident in my ability to learn and develop new skills relevant to my job.”

### Personal Benefits

Data on personal benefit has been gathered using the personal benefits employees required from the change measures (Holt et al., 2007). There were three items on this composing scale. Examples of items include “I’m concerned about the advantage of the change.”

## Results

### The Background of Respondents

The study used descriptive analysis to discuss the background of the respondents. Table 2 shows that the majority of respondents were between the ages of 36–40 (28.96%), followed by those between 31–35 (23.11%), 26–30 (19.22%), >40 (14.76%), 21–25 (12.53%), ≤20 (1.39%). In terms of gender, male participants were higher than females, 61.55 and 38.44%, respectively. The majority of the respondents (51.25%) were degree holders, 19.77% had master’s degrees, 3.06% Ph.D. holders, 25.90% others. This result indicates that most of the respondents were capable of responding unbiased responsible and truthful information for the consent of the study. In addition, more of the participants had 11–15 years of work experience (34.54%), followed by those with 6–10 years (30.64%), 1–5 years (22.84%), and >15 years (11.97%). This result also helps the authors to have clear, accurate, detailed information regarding the study’s purpose. The demographic information of the respondents has summarized as follows in Table 2.

**TABLE 2:** Demographic characteristics of the participant.

Items	Options	Frequency	Percentage (%)
Gender	Male	221	61.55
	Female	138	38.44
	Total	359	100%
Age	≤20	5	1.39
	21–25	45	12.53
	26–30	69	19.22
	31–35	83	23.11
	36–40	104	28.96
	>40	53	14.76
	Total	359	100%
Education	Bachelor	184	51.25
	Masters	71	19.77
	Ph.D.	11	3.06
	Others	93	25.90
	Total	359	100%
Experience	1–5	82	22.84
	6–10	110	30.64
	11–15	124	34.54
	>15	43	11.97
	Total	359	100%

### Reliability and Validity Test

In this study, reliability and validity tests have been done through the previously validated and recommended findings depending on the study’s objectives. Before beginning statistical analysis, the researcher performs a reliability and validity test with AMOS software version 23.0 and SPPS version 25 to ensure that all questionnaire instruments are valid based on the significant value. Confirmatory factor analysis (CFA) is used to ensure unidimensionality, convergent validity, discriminant validity, and reliability. According to the estimation instrument results (Table 3), all item loadings from 0.773 to 0.972 were statistically significant (Hair et al., 2006). In addition, all latent constructs’ measurements were found to have high convergent validity. The Cronbach’s alpha of all variables is >0.70, significant in the reliability test (Kothari, 2004). The latent constructs’ composite reliabilities (CR) ranged between 0.953228 and 0.969847, >0.70 shows a satisfactory level of reliability (Brusset, 2016). Furthermore, AVE estimates ranged from 0.77204 to 0.914696, greater than the recommended values of alpha >0.50 (Bagozzi and Yi, 2012). This result implies that the estimated values of AVE are adequate for all determined variables.

**TABLE 3:** Validation of instruments and statistics.

Variables	Items	Factor loading	Cronbach’s alpha	CR	AVE
ORJ	ORJ1 ORJ2 ORJ3 ORJ4 ORJ5 ORJ6 ORJ7 ORJ8	0.890 0.879 0.904 0.898 0.890 0.836 0.881 0.849	0.958	0.964383	0.77204
POS	POS1 POS2 POS3 POS4 POS5 POS6	0.909 0.916 0.936 0.933 0.912 0.773	0.952	0.961492	0.806866
ROC	ROC1 ROC2 ROC3 ROC4 ROC5 ROC6	0.846 0.886 0.900 0.880 0.876 0.885	0.941	0.953228	0.772619
CA	CA1 CA2 CA3	0.944 0.968 0.949	0.950	0.967926	0.909587
MS	MS1 MS2 MS3	0.950 0.959 0.945	0.947	0.966217	0.905069
SE	SE1 SE2 SE3	0.949 0.972 0.948	0.953	0.969847	0.914696
PB	PB1 PB2 PB3	0.957 0.966 0.934	0.949	0.96699	0.90712

*ORJ, organizational justice; POS, perceived organizational support; ROC, readiness for organizational change; CA, change appropriateness; MS, managerial support; SE, self-efficiency; PB, personal benefit; CR, composite reliability; AVE, average variance extracted.*

### Correlation Coefficient Matrix

Table 4 shows that organizational justice and POS are highly correlated with employee readiness for change. Four dimensions assess employee readiness to change. These are appropriateness, managerial support, self-efficiency, and personal benefit. Based on these dimensions, the correlation between employee readiness for change and the measurements is positive and significant, with a *p*-value of 0.01. The discriminant validity test was used to determine whether the value of the AVE square roots was more than the value of multiple correlation coefficients (Fornell and Larcker, 1981). As we can see from Table 4, the discriminant validity test shows a greater correlation matrix value for all variables and dimensions. Because of the result of discriminant validity, this study is supportive and acceptable.

**TABLE 4:** Means, standard deviations, and correlation coefficient matrix.

Variables	Mean	*SD*	ORJ	POS	ROC	CA	MS	*SE*	PB
ORJ	3.9638	0.84686	**0.879**						
POS	3.9749	0.87449	0.836**	**0.898**					
ROC	4.0682	0.76877	0.765**	0.830**	**0.879**				
CA	4.0669	0.81869	0.720**	0.740**	0.825**	**0.953**			
MS	4.1226	0.85102	0.742**	0.759**	0.827**	0.847**	**0.951**		
SE	4.0566	0.80706	0.682**	0.745**	0.788**	0.743**	0.791**	**0.985**	
PB	4.0947	0.80636	0.721**	0.776**	0.827**	0.786**	0.884**	0.957**	**0.972**

*ORJ, organizational justice; POS, perceived organizational support; ROC, readiness for organizational change; CA, change appropriateness; MS, managerial support; SE, self-efficiency; PB, personal benefit.*

Bold values are the square root of AVE, which indicates the discriminant validity test, and ** shows the correlation and level of significant among variables.

### The Model Fitness for Confirmatory Factor Analysis

The fit indices of the measurement model have shown in Table 5. GFI, AGFI, TLI, IFI, CFI, RMSEA, and x^2^/d. *f* were adequate using suggested values. Because of this, this model provided acceptance criteria for fit indices. The model fit indexes are used to determine the unidimensionality of each measurement construct. The model was estimated using GFI, AGFI, TLI, IFI, CFI, RMSEA and *x*^2^/d.f. The estimated model’s good fit was confirmed by the statistical values listed as follows: chi-square *x*^2^/d.f ≤ 3.0, GFI ≥ 0.9, AGFI ≥ 0.9, TLI ≥ 0.9, IFI ≥ 0.9, CFI ≥ 0.9 and RMSEA ≤ 0.08. Finally, the model fit (*x*^2^ = 201.031 d.f = 74; *X*^2^/d. *f* = 2.71; GFI = 0.929; AGFI = 0.899; TLI = 0.937; IFI = 0.952; CFI = 0.994; RMSEA 0.041) was adequate (Hair et al., 2006).

**TABLE 5:** Model fitness result for CFA.

Model	Criteria	Standard model
Chi-square (*X*^2^)		201.031
d.f		74
*X*^2^/d. f	≤3.0	2.71
GFI	≥0.9	0.929
AGFI	≥ 0.9	0.899
TLI	≥ 0.9	0.937
IFI	≥0.9	0.952
CFI	≥ 0.9	0.994
RMSEA	≤0.08	0.041

*x^2^/d.f, chi- square; GFI, goodness- of-fit index; AGFI, adjusted goodness of -fit index; TLI, Tucker-Lewis index; IFI, incremental fit index; CFI, comparative fit index; RMSEA, root mean square error of approximation.*

### Estimation of the Model and Testing of the Hypothesis

#### Estimation of Model

**FIGURE 2:**
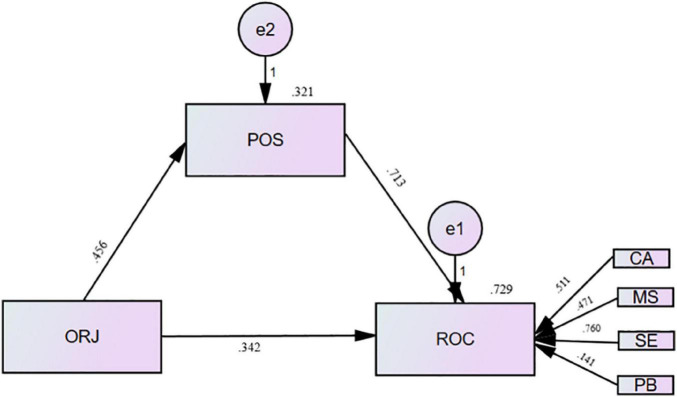
Standardized path coefficients and model significance.

In the structural equation model (SEM), the inner model represents the path structure between constructs. The results of hypothesis testing and path analysis based on the internal model have shown in Figure 2.

#### Testing of the Hypothesis

In this study, the derived hypothesis has been tested and identified aligned with designed research questions and specific objectives. Accordingly, the results and decisions have been indicated as follows.

**Hypothesis 1 (H_1_):** Organizational justice (ORJ) influenced employee readiness for organizational change (ROC) in a significant and positive way (β = 0.342, *p* < 0.01). As a result, the study supported hypothesis 1 (H_1_).

**Hypothesis 2 (H_2_):** ORJ influenced Perceived support (POS) significantly and positively (β = 0.456, *p* < 0.01). Based on this result, hypothesis 2 (H_2_) has been supported.

**Hypothesis 3 (H_3_):** POS had a statistically significant and positive effect on the Readiness of employees for organizational change (β = 0.713, *p* < 0.01). Hypothesis 3(H_3_) is also supported.

According to the result in Table 6, Hypothesis 1 (H_1_), Hypothesis 2 (H_2_), and Hypothesis 3 (H_3_) have been supported. This result indicates that all formulated hypotheses are accepted. The *R*^2^ of the estimated model shows that organizational justice and POS affect employee readiness for change by 72.9%, indicating that employee readiness for organizational change is affected by explained variables in the model. Unexplained factors have influenced the remaining 27.1%.

**TABLE 6:** Summarized results of the hypothesis.

Hypothesis	Path coefficient	*t*-value	Sig.	Inferences
**H_1_**: ORJ → ROC	0.342	4.564	0.000	Supported
**H_2_:** ORJ → POS	0.456	2.944	0.000	Supported
**H_3_:** POS → ROC	0.713	8.152	0.000	Supported
**Variance = *R*^2^**				
POS			ROC	
0.321			0.729	

*ORJ, organizational justice; POS, perceived organizational support; ROC, readiness for organizational change. p-value < 0.001.*

### Regression Analysis

This study used regression analysis to show the priorities of the variables which influence the dependent variable. The result of Table 7 shows that each factor estimate value ranging from 0.342 (ORJ to ROC), 0.456 (ORJ to POS), 0.713 (POS to ROC) has a direct impact between variables. On the other hand, the estimates range of four dimensions from 0.511 (CA), 0.471 (MS), 0.760 (SE), 0.141 (PB), to ROC has a significant influence on the dependent variable.

**TABLE 7:** Regression weights for the level of significant and critical ratio.

List of all variables path	Estimate	*SE*	C.R.	Sig
POS < — ORJ	0.456	0.017	2.944	0.000
ROC < — ORJ	0.342	0.055	4.564	0.000
ROC < — POS	0.713	0.066	8.152	0.000
ROC < — CA	0.511	0.051	2.588	0.000
ROC < — MS	0.471	0.063	3.508	0.000
ROC < — SE	0.760	0.057	2.734	0.000
ROC < — PB	0.141	0.048	3.054	0.000

*ORJ, organizational justice; POS, perceived organizational support; ROC, readiness for organizational change; CA, change appropriateness; MS, managerial support; SE, self-efficiency; PB, personal benefit; SE, standard error; C.R, critical ratio values.*

Critical ratio values (C.R) have generated when the estimates are separated by their relevant standard error (SE). C.R > 1.96 are thus statistically significant at the 0.01 level. All the C.R are greater than 2.588, indicating a significant level of significance. Furthermore, all study variables have been tested to ensure that the proposed research model fits its purpose. As a result, the predictor variable (ORJ) is positive and significant at a *p*-value of 0.01. The *p*-value for the mediator variable (POS) is 0.01, and the *p*-value for the dependent variable (ROC) is 0.01. This result indicates that the variances of variables are not significantly different across all dimensions.

### The Mediating Effect Analysis

In this study, the mediating effect has primarily been investigated using the steps established by Baron and Kenny (1986), validated and explored by the *z*-test (Sobel, 1982). The method proposed by MacKinnon et al. (2012) was also used in this study, which employed Sobel’s (1982) *z*-test (Tofighi and MacKinnon, 2011). The product distribution method calculated the confidence intervals for mediating effects during the analysis. In addition, all constructs were completed using mediating analysis. Table 6 summarizes the findings. To investigate the effect of the mediating variables, the Sobel-test was used. There are mediating effects when the *Z* score exceeds the *p*-value, which is greater than 0.05 (Sobel, 1982). For direct effects, the confidence intervals were calculated using the product distribution method. The confidence interval for direct effects did not include zero at a 95% confidence level, indicating that mediating effects were observed in this study. The mediating impact of POS in this study was statistically significant (Table 8).

**TABLE 8:** The mediating effect of perceived organizational support.

Mediator (Path)	Coefficient	Standard error	Sobel test *z*-value	LL95%CI	UL95%CI
ORJ → POS → ROC	0.456 0.713	0.017 0.066	10.02085011	0.348	0.544

*ORJ, organizational justice; POS, perceived organizational support; ROC, Readiness for organizational change.*

*p-value < 0.01.*

## Discussion

In this study, the author assesses the impact of organizational justice on the Readiness of employees for organizational change using POS as a mediator. According to the study’s findings, organizational justice and POS are important in preparing employees for organizational change (Mollahosseini et al., 2012). Therefore, managers and leaders must be concerned with these factors. If management intends to face change stakes, they should plan ahead of time by enhancing such organizational influences in a long time. Given the rapid pace of organizational change, management must treat their employees fairly (Lee et al., 2021). Therefore, to ensure the success of organizational change, ERCA’s leaders and managers must focus on how to support their employees by providing employees comfort, respecting employees’ opinions, motivating and assisting them in achieving their goals. Employees must also believe in the importance of change and should have confidence in their ability to adapt to it (Wanberg and Banas, 2000). As a result, this study shows the linkage between organizational justice and the Readiness of employees for organizational change is significant. This study also confirms that the relationship between organizational justice and the Readiness of employees for organizational change is significant with the mediation of POS.

Furthermore, this study investigated the dimensions of Readiness for Change to assess the impact of organizational justice on employee readiness for change. Accordingly, Organizational justice had a significant influence on all of these dimensions. These findings suggest that organizational justice positively influences Managerial support, Appropriateness, Self-efficacy, and Personal benefits and plays an important role in successfully implementing organizational change.

### Theoretical Contributions

This research contributes to the literature on organizational justice, organizational change, and change readiness. This study contributes to how to conduct successful organizational change by understanding the psychological behavior of employees during the change time. Overall, this study can also assist public and private organizations, as well as policymakers and practitioners, in improving organizational change practices by considering the positive effect of organizational justice on employee readiness for change and the mediating role of POS in the relationship between organizational justice and employee readiness for change.

### Practical Implications

The study suggests that ERCA should encourage its employees to contribute ideas for organizational change. As many studies stated, most organizational change initiatives are initiated by leaders/managers. Employees, for the most part, did not contribute ideas for change, which led to employee dissatisfaction and a lack of trust in the change that the organization deserved. According to the study’s findings, this approach is ineffective for successfully implementing organizational changes. Because employees from various departments are the most important performers in any activities, the organization should motivate them to generate or share ideas for improving their organization. This decision encourages employees to own the change while motivating and holding them accountable for their changing activities. Furthermore, before implementing the change, organizations must first understand what it is and how to implement it, as well as share lessons learned from institutions that have successfully implemented organizational changes. This assists leaders and managers of the ERCA and other related public and private organizations in understanding the factors influencing employee readiness for organizational change.

## Limitations and Recommendations for Future Studies

This research, like any other study, has limitations. Because of time limitations, this study is focused only on the relationship between organizational justices and employee readiness for change, using POS as a mediator. The authors recommend further studying other factors detrimental to employee readiness for change. For example, factors like participation in the change process (Wanberg and Banas, 2000), change communication (Miller and Monge, 1985), and coping with change (Amiot et al., 2006). In addition, the survey included only respondents from ERCA’s one branch rather than from all ERCA branches across the country. However, the goal is to start discussions about future directions. Therefore, potential factors can be considered in future research.

## Conclusion

The study investigated the influence of organizational justice on employee readiness for change and considered the mediating role of POS. According to the findings, organizational justice has a positive and significant impact on employees’ Readiness for change. Organizational justice positively relates with all selected dimensions of change readiness (change appropriateness, managerial support, self-efficiency, and personal benefit). In addition, the study shows there is a positive relationship between organizational justice and POS. Furthermore, the study discovered that the influence of organizational justice on the Readiness of employees for organizational change via POS is also significant. Even though the literature indicates that ERCA has not fully implemented the successful organizational changes, the findings are significant. According to the findings of the study’s, it is critical to assess the influence of organizational justice on employee readiness for change to run a successful organizational change.

## Data Availability Statement

The raw data supporting the conclusions of this article will be made available by the authors, without undue reservation.

## Ethics Statement

Ethical review and approval was not required for the study on human participants in accordance with the local legislation and institutional requirements. Written informed consent for participation was not required for this study in accordance with the national legislation and the institutional requirements.

## Author Contributions

AW and SK: conceptualization and methodology. SK: software, validation, data collection, formal analysis, investigation, and writing the original draft preparation. AW: review, editing, and supervision. Both authors read and agreed to the published version of the manuscript.

## Conflict of Interest

The authors declare that the research was conducted in the absence of any commercial or financial relationships that could be construed as a potential conflict of interest.

## Publisher’s Note

All claims expressed in this article are solely those of the authors and do not necessarily represent those of their affiliated organizations, or those of the publisher, the editors and the reviewers. Any product that may be evaluated in this article, or claim that may be made by its manufacturer, is not guaranteed or endorsed by the publisher.
